# Making sense of implementation theories, models and frameworks

**DOI:** 10.1186/s13012-015-0242-0

**Published:** 2015-04-21

**Authors:** Per Nilsen

**Affiliations:** Division of Community Medicine, Department of Medical and Health Sciences, Linköping University, Linköping, Sweden

**Keywords:** Theory, Model, Framework, Evaluation, Context

## Abstract

**Background:**

Implementation science has progressed towards increased use of theoretical approaches to provide better understanding and explanation of how and why implementation succeeds or fails. The aim of this article is to propose a taxonomy that distinguishes between different categories of theories, models and frameworks in implementation science, to facilitate appropriate selection and application of relevant approaches in implementation research and practice and to foster cross-disciplinary dialogue among implementation researchers.

**Discussion:**

Theoretical approaches used in implementation science have three overarching aims: describing and/or guiding the process of translating research into practice (process models); understanding and/or explaining what influences implementation outcomes (determinant frameworks, classic theories, implementation theories); and evaluating implementation (evaluation frameworks).

**Summary:**

This article proposes five categories of theoretical approaches to achieve three overarching aims. These categories are not always recognized as separate types of approaches in the literature. While there is overlap between some of the theories, models and frameworks, awareness of the differences is important to facilitate the selection of relevant approaches. Most determinant frameworks provide limited “how-to” support for carrying out implementation endeavours since the determinants usually are too generic to provide sufficient detail for guiding an implementation process. And while the relevance of addressing barriers and enablers to translating research into practice is mentioned in many process models, these models do not identify or systematically structure specific determinants associated with implementation success. Furthermore, process models recognize a temporal sequence of implementation endeavours, whereas determinant frameworks do not explicitly take a process perspective of implementation.

## Background

Implementation science was borne out of a desire to address challenges associated with the use of research to achieve more evidence-based practice (EBP) in health care and other areas of professional practice. Early implementation research was empirically driven and did not always pay attention to the theoretical underpinnings of implementation. Eccles et al. ([1]:108) remarked that this research seemed like “an expensive version of trial-and-error”. A review of guideline implementation strategies by Davies et al. [2] noted that only 10% of the studies identified provided an explicit rationale for their strategies. Mixed results of implementing EBP in various settings were often attributed to a limited theoretical basis [1,3-5]. Poor theoretical underpinning makes it difficult to understand and explain how and why implementation succeeds or fails, thus restraining opportunities to identify factors that predict the likelihood of implementation success and develop better strategies to achieve more successful implementation.

However, the last decade of implementation science has seen wider recognition of the need to establish the theoretical bases of implementation and strategies to facilitate implementation. There is mounting interest in the use of theories, models and frameworks to gain insights into the mechanisms by which implementation is more likely to succeed. Implementation studies now apply theories borrowed from disciplines such as psychology, sociology and organizational theory as well as theories, models and frameworks that have emerged from within implementation science. There are now so many theoretical approaches that some researchers have complained about the difficulties of choosing the most appropriate [6-11].

This article seeks to further implementation science by providing a narrative review of the theories, models and frameworks applied in this research field. The aim is to describe and analyse how theories, models and frameworks have been applied in implementation science and propose a taxonomy that distinguishes between different approaches to advance clarity and achieve a common terminology. The ambition is to facilitate appropriate selection and application of relevant approaches in implementation studies and foster cross-disciplinary dialogue among implementation researchers. The importance of a clarifying taxonomy has evolved during the many discussions on theoretical approaches used within implementation science that the author has had over the past few years with fellow implementation researchers, as well as reflection on the utility of different approaches in various situations.

Implementation science is defined as the scientific study of methods to promote the systematic uptake of research findings and other EBPs into routine practice to improve the quality and effectiveness of health services and care [12]. The terms knowledge translation, knowledge exchange, knowledge transfer, knowledge integration and research utilization are used to describe overlapping and interrelated research on putting various forms of knowledge, including research, to use [8,13-16]. Implementation is part of a diffusion-dissemination-implementation continuum: diffusion is the passive, untargeted and unplanned spread of new practices; dissemination is the active spread of new practices to the target audience using planned strategies; and implementation is the process of putting to use or integrating new practices within a setting [16,17].

A narrative review of selective literature was undertaken to identify key theories, models and frameworks used in implementation science. The narrative review approach gathers information about a particular subject from many sources and is considered appropriate for summarizing and synthesizing the literature to draw conclusions about “what we know” about the subject. Narrative reviews yield qualitative results, with strengths in capturing diversities and pluralities of understanding [18,19]. Six textbooks that provide comprehensive overviews of research regarding implementation science and implementation of EBP were consulted: Rycroft-Malone and Bucknall [20], Nutley et al. [21], Greenhalgh et al. [17], Grol et al. [22], Straus et al. [23] and Brownson et al. [24]. A few papers presenting overviews of theories, models and frameworks used in implementation science were also used: Estabrooks et al. [14], Sales et al. [4], Graham and Tetroe [25], Mitchell et al. [8], Flottorp et al. [26], Meyers et al. [27] and Tabak et al. [28]. In addition, *Implementation Science* (first published in 2006) was searched using the terms “theory”, “model” and “framework” to identify relevant articles. The titles and abstracts of the identified articles were scanned, and those that were relevant to the study aim were read in full.

## Discussion

### Theories, models and frameworks in the general literature

Generally, a theory may be defined as a set of analytical principles or statements designed to structure our observation, understanding and explanation of the world [29-31]. Authors usually point to a theory as being made up of definitions of variables, a domain where the theory applies, a set of relationships between the variables and specific predictions [32-35]. A “good theory” provides a clear explanation of how and why specific relationships lead to specific events. Theories can be described on an abstraction continuum. High abstraction level theories (general or grand theories) have an almost unlimited scope, middle abstraction level theories explain limited sets of phenomena and lower level abstraction theories are empirical generalizations of limited scope and application [30,36].

A model typically involves a deliberate simplification of a phenomenon or a specific aspect of a phenomenon. Models need not be completely accurate representations of reality to have value [31,37]. Models are closely related to theory and the difference between a theory and a model is not always clear. Models can be described as theories with a more narrowly defined scope of explanation; a model is descriptive, whereas a theory is explanatory as well as descriptive [29].

A framework usually denotes a structure, overview, outline, system or plan consisting of various descriptive categories, e.g. concepts, constructs or variables, and the relations between them that are presumed to account for a phenomenon [38]. Frameworks do not provide explanations; they only describe empirical phenomena by fitting them into a set of categories [29].

### Theories, models and frameworks in implementation science

**Figure 1:**
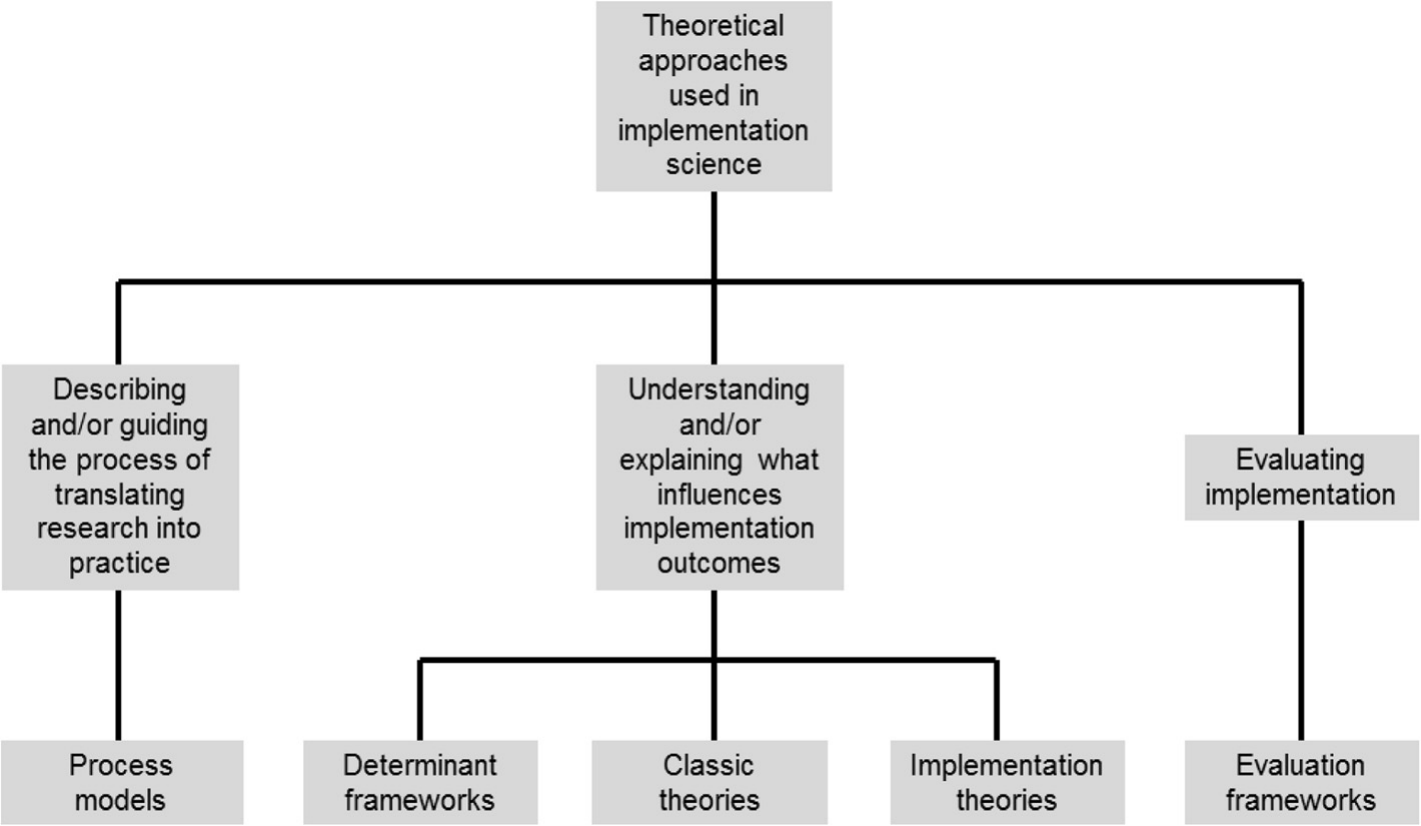
Three aims of the use of theoretical approaches in implementation science and the five categories of theories, models and frameworks.

It was possible to identify three overarching aims of the use of theories, models and frameworks in implementation science: (1) describing and/or guiding the process of translating research into practice, (2) understanding and/or explaining what influences implementation outcomes and (3) evaluating implementation. Theoretical approaches which aim at understanding and/or explaining influences on implementation outcomes (i.e. the second aim) can be further broken down into determinant frameworks, classic theories and implementation theories based on descriptions of their origins, how they were developed, what knowledge sources they drew on, stated aims and applications in implementation science. Thus, five categories of theoretical approaches used in implementation science can be delineated (Table 1; Figure 1):

**Table 1:** **Five categories of theories, models and frameworks used in implementation science**

**Category**	**Description**	**Examples**
Process models	Specify steps (stages, phases) in the process of translating research into practice, including the implementation and use of research. The aim of process models is to describe and/or guide the process of translating research into practice. An action model is a type of process model that provides practical guidance in the planning and execution of implementation endeavours and/or implementation strategies to facilitate implementation. Note that the terms “model” and “framework” are both used, but the former appears to be the most common	Model by Huberman [40], model by Landry et al. [41], model by Davies et al. [43], model by Majdzadeh et al. [44], the CIHR Model of Knowledge Translation [42], the K2A Framework [15], the Stetler Model [47], the ACE Star Model of Knowledge Transformation [48], the Knowledge-to-Action Model [13], the Iowa Model [49,50], the Ottawa Model [51,52], model by Grol and Wensing [53], model by Pronovost et al. [54], the Quality Implementation Framework [27]
Determinant frameworks	Specify types (also known as classes or domains) of determinants and individual determinants, which act as barriers and enablers (independent variables) that influence implementation outcomes (dependent variables). Some frameworks also specify relationships between some types of determinants. The overarching aim is to understand and/or explain influences on implementation outcomes, e.g. predicting outcomes or interpreting outcomes retrospectively	PARIHS [5,64], Active Implementation Frameworks [63,68], Understanding-User-Context Framework [62], Conceptual Model [17], framework by Grol et al. [22], framework by Cochrane et al. [59], framework by Nutley et al. [21], Ecological Framework by Durlak and DuPre [57], CFIR [60], framework by Gurses et al. [58], framework by Ferlie and Shortell [61], Theoretical Domains Framework [66]
Classic theories	Theories that originate from fields external to implementation science, e.g. psychology, sociology and organizational theory, which can be applied to provide understanding and/or explanation of aspects of implementation	Theory of Diffusion [107], social cognitive theories, theories concerning cognitive processes and decision making, social networks theories, social capital theories, communities of practice, professional theories, organizational theories
Implementation theories	Theories that have been developed by implementation researchers (from scratch or by adapting existing theories and concepts) to provide understanding and/or explanation of aspects of implementation	Implementation Climate [116], Absorptive Capacity [117], Organizational Readiness [118], COM-B [119], Normalization Process Theory [120]
Evaluation frameworks	Specify aspects of implementation that could be evaluated to determine implementation success	RE-AIM [124]; PRECEDE-PROCEED [125]; framework by Proctor et al. [126]

*ACE* Academic Center for Evidence-Based Practice, *CFIR* Consolidated Framework for Implementation Research, *CIHR* Canadian Institutes of Health Research Knowledge, *COM-B* Capacity-Opportunities-Motivation-Behaviour, *Conceptual Model* Conceptual Model for Considering the Determinants of Diffusion, Dissemination, and Implementation of Innovations in Health Service Delivery and Organization (full title), *K2A* Knowledge-to-Action, *PARIHS* Promoting Action on Research Implementation in Health Services, *PRECEDE-PROCEED* Predisposing, Reinforcing and Enabling Constructs in Educational Diagnosis and Evaluation-Policy, Regulatory, and Organizational Constructs in Educational and Environmental Development, *RE-AIM* Reach, Effectiveness, Adoption, Implementation, Maintenance.

- Process models
- Determinant frameworks
- Classic theories
- Implementation theories
- Evaluation frameworks

Although theories, models and frameworks are distinct concepts, the terms are sometimes used interchangeably in implementation science [9,14,39]. A theory in this field usually implies some predictive capacity (e.g. to what extent do health care practitioners’ attitudes and beliefs concerning a clinical guideline predict their adherence to this guideline in clinical practice?) and attempts to explain the causal mechanisms of implementation. Models in implementation science are commonly used to describe and/or guide the process of translating research into practice (i.e. “implementation practice”) rather than to predict or analyse what factors influence implementation outcomes (i.e. “implementation research”). Frameworks in implementation science often have a descriptive purpose by pointing to factors believed or found to influence implementation outcomes (e.g. health care practitioners’ adoption of an evidence-based patient intervention). Neither models nor frameworks specify the mechanisms of change; they are typically more like checklists of factors relevant to various aspects of implementation.

### Describing and/or guiding the process of translating research into practice

#### Process models

Process models are used to describe and/or guide the process of translating research into practice. Models by Huberman [40], Landry et al. [41], the CIHR (Canadian Institutes of Health Research) Knowledge Model of Knowledge Translation [42], Davis et al. [43], Majdzadeh et al. [44] and the K2A (Knowledge-to-Action) Framework [15] outline phases or stages of the research-to-practice process, from discovery and production of research-based knowledge to implementation and use of research in various settings.

Early research-to-practice (or knowledge-to-action) models tended to depict rational, linear processes in which research was simply transferred from producers to users. However, subsequent models have highlighted the importance of facilitation to support the process and placed more emphasis on the contexts in which research is implemented and used. Thus, the attention has shifted from a focus on production, diffusion and dissemination of research to various implementation aspects [21].

So-called action (or planned action) models are process models that facilitate implementation by offering practical guidance in the planning and execution of implementation endeavours and/or implementation strategies. Action models elucidate important aspects that need to be considered in implementation practice and usually prescribe a number of stages or steps that should be followed in the process of translating research into practice. Action models have been described as active by Graham et al. ([45]:185) because they are used “to guide or cause change”. It should be noted that the terminology is not fully consistent, as some of these models are referred to as frameworks, for instance the Knowledge-to-Action Framework [46].

Many of the action models originate from the nursing-led field of research use/utilization; well-known examples include the Stetler Model [47], the ACE (Academic Center for Evidence-Based Practice) Star Model of Knowledge Transformation [48], the Knowledge-to-Action Framework [13], the Iowa Model [49,50] and the Ottawa Model [51,52]. There are also numerous examples of similar “how-to-implement” models that have emerged from other fields, including models developed by Grol and Wensing [53], Pronovost et al. [54] and the Quality Implementation Framework [27], all of which are intended to provide support for planning and managing implementation endeavours.

The how-to-implement models typically emphasize the importance of careful, deliberate planning, especially in the early stages of implementation endeavours. In many ways, they present an ideal view of implementation practice as a process that proceeds step-wise, in an orderly, linear fashion. Still, authors behind most models emphasize that the actual process is not necessarily sequential. Many of the action models mentioned here have been subjected to testing or evaluation, and some have been widely applied in empirical research, underscoring their usefulness [9,55].

The process models vary with regard to how they were developed. Models such as the Stetler Model [47,56] and the Iowa Model [49,50] were based on the originators’ own experiences of implementing new practices in various settings (although they were also informed by research and expert opinion). In contrast, models such as the Knowledge-to-Action Framework [45] and the Quality Implementation Framework [27] have relied on literature reviews of theories, models, frameworks and individual studies to identify key features of successful implementation endeavours.

### Understanding and explaining what influences implementation outcomes

#### Determinant frameworks

Determinant frameworks describe general types (also referred to as classes or domains) of determinants that are hypothesized or have been found to influence implementation outcomes, e.g. health care professionals’ behaviour change or adherence to a clinical guideline. Each type of determinant typically comprises a number of individual barriers (hinders, impediments) and/or enablers (facilitators), which are seen as independent variables that have an impact on implementation outcomes, i.e. the dependent variable. Some frameworks also hypothesize relationships between these determinants (e.g. [17,57,58]), whereas others recognize such relationships without clarifying them (e.g. [59,60]). Information about what influences implementation outcomes is potentially useful for designing and executing implementation strategies that aim to change relevant determinants.

The determinant frameworks do not address how change takes place or any causal mechanisms, underscoring that they should not be considered theories. Many frameworks are multilevel, identifying determinants at different levels, from the individual user or adopter (e.g. health care practitioners) to the organization and beyond. Hence, these integrative frameworks recognize that implementation is a multidimensional phenomenon, with multiple interacting influences.

The determinant frameworks were developed in different ways. Many frameworks (e.g. [17,21,22,57,59,61]) were developed by synthesizing results from empirical studies of barriers and enablers for implementation success. Other frameworks have relied on existing determinant frameworks and relevant theories in various disciplines, e.g. the frameworks by Gurses et al. [58] and CFIR (Consolidated Framework for Implementation Research) [60].

Several frameworks have drawn extensively on the originator’s own experiences of implementing new practices. For instance, the Understanding-User-Context Framework [62] and Active Implementation Frameworks [63] were both based on a combination of literature reviews and the originators’ implementation experiences. Meanwhile, PARIHS (Promoting Action on Research Implementation in Health Services) [5,64] emerged from the observation that successful implementation in health care might be premised on three key determinants (characteristics of the evidence, context and facilitation), a proposition which was then analysed in four empirical case studies; PARIHS has subsequently undergone substantial research and development work [64] and has been widely applied [65].

Theoretical Domains Framework represents another approach to developing determinant frameworks. It was constructed on the basis of a synthesis of 128 constructs related to behaviour change found in 33 behaviour change theories, including many social cognitive theories [10]. The constructs are sorted into 14 theoretical domains (originally 12 domains), e.g. knowledge, skills, intentions, goals, social influences and beliefs about capabilities [66]. Theoretical Domains Framework does not specify the causal mechanisms found in the original theories, thus sharing many characteristics with determinant frameworks.

The determinant frameworks account for five types of determinants, as shown in Table 2, which provides details of eight of the most commonly cited frameworks in implementation science. The frameworks are superficially quite disparate, with a broad range of terms, concepts and constructs as well as different outcomes, yet they are quite similar with regard to the general types of determinants they account for. Hence, implementation researchers agree to a large extent on what the main influences on implementation outcomes are, albeit to a lesser extent on which terms that are best used to describe these determinants.

**Table 2:** **Implementation determinants and outcomes in eight determinant frameworks**

	**Characteristics of the implementation object**	**Characteristics of the users/adopters (e.g. health care practitioners)**	**Characteristics of the end users (e.g. patients)**	**Characteristics of the context**	**Characteristics of the strategy or other means of facilitating implementation**	**Outcomes**
PARIHS (Kitson et al. [5]; Rycroft-Malone [64])	Characteristics of the evidence	Characteristics of the clinical experience (addressed as an aspect of the evidence element)	Characteristics of the patient experience (addressed as an aspect of the evidence element)	Characteristics of the context (comprising culture, leadership and evaluation)	Characteristics of the facilitation, i.e. the process of enabling or making easier the implementation	Successful implementation of research
Conceptual Model (Greenhalgh et al. [17])	Innovation attributes	Aspects of adopters (e.g. psychological antecedents and nature of the adoption decision) and assimilation by organizations	Not addressed	Features of the inner context (organizational antecedents and organizational readiness for innovation) and outer context (e.g. informal interorganizational networks and political directives)	Influences (e.g. opinion leaders, champions and network structure) lying on a continuum from diffusion to dissemination	Successful diffusion, dissemination and implementation of innovations
Grol et al. [22]	Features of the innovation	Features of the professionals who should use the innovation	Features of the patients	Features of the social setting (e.g. attitudes of colleagues, culture and leadership) and of the economic, administrative and organizational context	Features of the methods and strategies for dissemination and implementation used	Implementation of new evidence, guidelines and best practices or procedures
Nutley et al. [21]	Nature of the research to be applied	Personal characteristics of researchers and potential research users and links between research and its users	Not addressed	Context for the use of research	Strategies to improve the use of research	Use of research
Cochrane et al. [59]	Guidelines and evidence barriers	Cognitive and behavioural barriers, attitudinal and rational-emotional barriers, health care professional and physician barriers	Patient barriers	Support and resource barriers, system and process barriers	Not addressed	Adherence to guidelines or implementation of evidence into clinical practice
Ecological Framework (Durlak and DuPre [57])	Characteristics of the innovation	Provider characteristics	Not addressed	Community-level factors (comprising general organizational features, specific organizational practices and processes, and specific staffing considerations)	Features of the prevention support system (comprising training and technical assistance)	Successful implementation of innovations
CFIR (Damschroder et al. [60])	Intervention characteristics	Characteristics of individuals	Patient needs and resources (addressed as an aspect of the outer setting)	Characteristics of the inner setting (e.g. structural characteristics, networks and communications, culture) and outer setting (e.g. cosmopolitanism, external policies and incentives)	Effectiveness of process by which implementation is accomplished (comprising planning, engaging, executing, reflection and evaluating)	Successful implementation of interventions
Gurses et al. [58]	Guideline characteristics	Clinician characteristics	Not addressed	Systems characteristics (e.g. physical environment, organizational characteristics) and implementation characteristics (e.g. tension for change and getting ideas from outside the organization)	Implementation characteristics (e.g. change agents’ characteristics, relative strengths of supporters and opponents)	Adherence to guidelines

*Conceptual Model* Conceptual Model for Considering the Determinants of Diffusion, Dissemination, and Implementation of Innovations in Health Service Delivery and Organization (full title, [17]), *CFIR* Consolidated Framework for Implementation Research, *PARIHS* Promoting Action on Research Implementation in Health Services.

The frameworks describe “implementation objects” in terms of research, guidelines, interventions, innovations and evidence (i.e. research-based knowledge in a broad sense). Outcomes differ correspondingly, from adherence to guidelines and research use, to successful implementation of interventions, innovations, evidence, etc. (i.e. the application of research-based knowledge in practice). The relevance of the end users (e.g. patients, consumers or community populations) of the implemented object (e.g. an EBP) is not explicitly addressed in some frameworks (e.g. [17,21,67]), suggesting that this is an area where further research is needed for better analysis of how various end users may influence implementation effectiveness.

Determinant frameworks imply a systems approach to implementation because they point to multiple levels of influence and acknowledge that there are relationships within and across the levels and different types of determinants. A system can be understood only as an integrated whole because it is composed not only of the sum of its components but also by the relationships among those components [68]. However, determinants are often assessed individually in implementation studies (e.g. [69-72]), (implicitly) assuming a linear relationship between the determinants and the outcomes and ignoring that individual barriers and enablers may interact in various ways that can be difficult to predict. For instance, there could be synergistic effects such that two seemingly minor barriers constitute an important obstacle to successful outcomes if they interact. Another issue is whether all relevant barriers and enablers are examined in these studies, which are often based on survey questionnaires, and are thus biased by the researcher’s selection of determinants. Surveying the perceived importance of a finite set of predetermined barriers can yield insights into the relative importance of these particular barriers but may overlook factors that independently affect implementation outcomes. Furthermore, there is the issue of whether the barriers and enablers are the actual determinants (i.e. whether they have actually been experienced or encountered) and the extent to which they are perceived to exist (i.e. they are more hypothetical barriers and enablers). The perceived importance of particular factors may not always correspond with the actual importance.

The context is an integral part of all the determinant frameworks. Described as “an important but poorly understood mediator of change and innovation in health care organizations” ([73]:79), the context lacks a unifying definition in implementation science (and related fields such as organizational behaviour and quality improvement). Still, context is generally understood as the conditions or surroundings in which something exists or occurs, typically referring to an analytical unit that is higher than the phenomena directly under investigation. The role afforded the context varies, from studies (e.g. [74-77]) that essentially view the context in terms of a physical “environment or setting in which the proposed change is to be implemented” ([5]:150) to studies (e.g. [21,74,78]) that assume that the context is something more active and dynamic that greatly affects the implementation process and outcomes. Hence, although implementation science researchers agree that the context is a critically important concept for understanding and explaining implementation, there is a lack of consensus regarding how this concept should be interpreted, in what ways the context is manifested and the means by which contextual influences might be captured in research.

The different types of determinants specified in determinant frameworks can be linked to classic theories. Thus, psychological theories that delineate factors influencing individual behaviour change are relevant for analysing how user/adopter characteristics affect implementation outcomes, whereas organizational theories concerning organizational climate, culture and leadership are more applicable for addressing the influence of the context on implementation outcomes.

#### Classic theories

Implementation researchers are also wont to apply theories from other fields such as psychology, sociology and organizational theory. These theories have been referred to as classic (or classic change) theories to distinguish them from research-to-practice models [45]. They might be considered passive in relation to action models because they describe change mechanisms and explain how change occurs without ambitions to actually bring about change.

Psychological behaviour change theories such as the Theory of Reasoned Action [79], the Social Cognitive Theory [80,81], the Theory of Interpersonal Behaviour [82] and the Theory of Planned Behaviour [83] have all been widely used in implementation science to study determinants of “clinical behaviour” change [84]. Theories such as the Cognitive Continuum Theory [85], the Novice-Expert Theory [86], the Cognitive-Experiential Self-Theory [87] and habit theories (e.g. [88,89]) may also be applicable for analysing cognitive processes involved in clinical decision-making and implementing EBP, but they are not as extensively used as the behaviour change theories.

Theories regarding the collective (such as health care teams) or other aggregate levels are relevant in implementation science, e.g. theories concerning professions and communities of practice, as well as theories concerning the relationships between individuals, e.g. social networks and social capital [14,53,90-93]. However, their use is not as prevalent as the individual-level theories.

There is increasing interest among implementation researchers in using theories concerning the organizational level because the context of implementation is becoming more widely acknowledged as an important influence on implementation outcomes. Theories concerning organizational culture, organizational climate, leadership and organizational learning are relevant for understanding and explaining organizational influences on implementation processes [21,53,57,94-101]. Several organization-level theories might have relevance for implementation science. For instance, Estabrooks et al. [14] have proposed the use of the Situated Change Theory [102] and the Institutional Theory [103,104], whereas Plsek and Greenhalgh [105] have suggested the use of complexity science [106] for better understanding of organizations. Meanwhile, Grol et al. [22] have highlighted the relevance of economic theories and theories of innovative organizations. However, despite increased interest in organizational theories, their actual use in empirical implementation studies thus far is relatively limited.

The Theory of Diffusion, as popularized through Rogers’ work on the spread of innovations, has also influenced implementation science. The theory’s notion of innovation attributes, i.e. relative advantage, compatibility, complexity, trialability and observability [107], has been widely applied in implementation science, both in individual studies (e.g. [108-110]) and in determinant frameworks (e.g. [17,58,60]) to assess the extent to which the characteristics of the implementation object (e.g. a clinical guideline) affect implementation outcomes. Furthermore, the Theory of Diffusion highlights the importance of intermediary actors (opinion leaders, change agents and gate-keepers) for successful adoption and implementation [107], which is reflected in roles described in numerous implementation determinant frameworks (e.g. [63,64]) and implementation strategy taxonomies (e.g. [111-114]). The Theory of Diffusion is considered the single most influential theory in the broader field of knowledge utilization of which implementation science is a part [115].

#### Implementation theories

There are also numerous theories that have been developed or adapted by researchers for potential use in implementation science to achieve enhanced understanding and explanation of certain aspects of implementation. Some of these have been developed by modifying certain features of existing theories or concepts, e.g. concerning organizational climate and culture. Examples include theories such as Implementation Climate [116], Absorptive Capacity [117] and Organizational Readiness [118]. The adaptation allows researchers to prioritize aspects considered to be most critical to analyse issues related to the how and why of implementation, thus improving the relevance and appropriateness to the particular circumstances at hand.

COM-B (Capability, Opportunity, Motivation and Behaviour) represents another approach to developing theories that might be applicable in implementation science. This theory began by identifying motivation as a process that energizes and directs behaviour. Capability and opportunity were added as necessary conditions for a volitional behaviour to occur, given sufficient motivation, on the basis of a US consensus meeting of behavioural theorists and a principle of US criminal law (which considers prerequisites for performance of specified volitional behaviours) [119]. COM-B posits that capability, opportunity and motivation generate behaviour, which in turn influences the three components. Opportunity and capability can influence motivation, while enacting a behaviour can alter capability, motivation and opportunity [66].

Another theory used in implementation science, the Normalization Process Theory [120], began life as a model, constructed on the basis of empirical studies of the implementation of new technologies [121]. The model was subsequently expanded upon and developed into a theory as change mechanisms and interrelations between various constructs were delineated [122]. The theory identifies four determinants of embedding (i.e. normalizing) complex interventions in practice (coherence or sense making, cognitive participation or engagement, collective action and reflexive monitoring) and the relationships between these determinants [123].

### Evaluating implementation

#### Evaluation frameworks

There is a category of frameworks that provide a structure for evaluating implementation endeavours. Two common frameworks that originated in public health are RE-AIM (Reach, Effectiveness, Adoption, Implementation, Maintenance) [124] and PRECEDE-PROCEED (Predisposing, Reinforcing and Enabling Constructs in Educational Diagnosis and Evaluation-Policy, Regulatory, and Organizational Constructs in Educational and Environmental Development) [125]. Both frameworks specify implementation aspects that should be evaluated as part of intervention studies.

Proctor et al. [126] have developed a framework of implementation outcomes that can be applied to evaluate implementation endeavours. On the basis of a narrative literature review, they propose eight conceptually distinct outcomes for potential evaluation: acceptability, adoption (also referred to as uptake), appropriateness, costs, feasibility, fidelity, penetration (integration of a practice within a specific setting) and sustainability (also referred to as maintenance or institutionalization).

Although evaluation frameworks may be considered in a category of their own, theories, models and frameworks from the other four categories can also be applied for evaluation purposes because they specify concepts and constructs that may be operationalized and measured. For instance, Theoretical Domains Framework (e.g. [127,128]), and Normalization Process Theory [129] and COM-B (e.g. [130,131]) have all been widely used as evaluation frameworks. Furthermore, many theories, models and frameworks have spawned instruments that serve evaluation purposes, e.g. tools linked to PARIHS [132,133], CFIR [134] and Theoretical Domains Framework [135]. Other examples include the EBP Implementation Scale to measure the extent to which EBP is implemented [136] and the BARRIERS Scale to identify barriers to research use [137], as well as instruments to operationalize theories such as Implementation Climate [138] and Organizational Readiness [139].

## Summary

Implementation science has progressed towards increased use of theoretical approaches to address various implementation challenges. While this article is not intended as a complete catalogue of all individual approaches available in implementation science, it is obvious that the menu of potentially useable theories, models and frameworks is extensive. Researchers in the field have pragmatically looked into other fields and disciplines to find relevant approaches, thus emphasizing the interdisciplinary and multiprofessional nature of the field.

This article proposes a taxonomy of five categories of theories, models and frameworks used in implementation science. These categories are not always recognized as separate types of approaches in the literature. For instance, systematic reviews and overviews by Graham and Tetroe [25], Mitchell et al. [8], Flottorp et al. [26], Meyers et al. [27] and Tabak et al. [28] have not distinguished between process models, determinant frameworks or classic theories because they all deal with factors believed or found to have an impact on implementation processes and outcomes. However, what matters most is not how an individual approach is labelled; it is important to recognize that these theories, models and frameworks differ in terms of their assumptions, aims and other characteristics, which have implications for their use.

There is considerable overlap between some of the categories. Thus, determinant frameworks, classic theories and implementation theories can also help to guide implementation practice (i.e. functioning as action models), because they identify potential barriers and enablers that might be important to address when undertaking an implementation endeavour. They can also be used for evaluation because they describe aspects that might be important to evaluate. A framework such as the Active Implementation Frameworks [68] appears to have a dual aim of providing hands-on support to implement something and identifying determinants of this implementation that should be analysed. Somewhat similarly, PARIHS [5] can be used by “anyone either attempting to get evidence into practice, or anyone who is researching or trying to better understand implementation processes and influences” ([64]:120), suggesting that it has ambitions that go beyond its primary function as a determinant framework.

Despite the overlap between different theories, models and frameworks used in implementation science, knowledge about the three overarching aims and five categories of theoretical approaches is important to identify and select relevant approaches in various situations. Most determinant frameworks provide limited “how-to” support for carrying out implementation endeavours since the determinants may be too generic to provide sufficient detail for guiding users through an implementation process. While the relevance of addressing barriers and enablers to translating research into practice is mentioned in many process models, these models do not identify or systematically structure specific determinants associated with implementation success. Another key difference is that process models recognize a temporal sequence of implementation endeavours, whereas determinant frameworks do not explicitly take a process perspective of implementation since the determinants typically relate to implementation as a whole.

Theories applied in implementation science can be characterized as middle level. Higher level theories can be built from theories at lower abstraction levels, so-called theory ladder climbing [140]. May [141] has discussed how a “general theory of implementation” might be constructed by linking the four constructs of Normalization Process Theory with constructs from relevant sociology and psychology theories to provide a more comprehensive explanation of the constituents of implementation processes. Still, it seems unlikely that there will ever be a grand implementation theory since implementation is too multifaceted and complex a phenomenon to allow for universal explanations. There has been debate in the policy implementation research field for many years whether researchers should strive to produce a theory applicable to public policy as a whole [38]. However, policy implementation researchers have increasingly argued that it would be a futile undertaking because “the world is too complex and there are too many causes of outcomes to allow for parsimonious explanation” ([37]:31). Determinant frameworks in implementation science clearly suggest that many different theories are relevant for understanding and explaining the many influences on implementation.

The use of a single theory that focuses only on a particular aspect of implementation will not tell the whole story. Choosing one approach often means placing weight on some aspects (e.g. certain causal factors) at the expense of others, thus offering only partial understanding. Combining the merits of multiple theoretical approaches may offer more complete understanding and explanation, yet such combinations may mask contrasting assumptions regarding key issues. For instance, are people driven primarily by their individual beliefs and motivation or does a pervasive organizational culture impose norms and values that regulate how people behave and make individual characteristics relatively unimportant? Is a particular behaviour primarily influenced by reflective thought processes or is it an automatically enacted habit? Furthermore, different approaches may require different methods, based on different epistemological and ontological assumptions.

There is a current wave of optimism in implementation science that using theoretical approaches will contribute to reducing the research-practice gap [4,10,11,63,142]. Although the use of theories, models and frameworks has many advocates in implementation science, there have also been critics [143,144], who have argued that theory is not necessarily better than common sense for guiding implementation. Common sense has been defined as a group’s shared tacit knowledge concerning a phenomenon [145]. One could argue that common sense about how or why something works (or does not) also constitutes a theory, albeit an informal and non-codified one. In either case, empirical research is needed to study how and the extent to which the use of implementation theories, models and frameworks contributes to more effective implementation and under what contextual conditions or circumstances they apply (and do not apply). It is also important to explore how the current theoretical approaches can be further developed to better address implementation challenges. Hence, both inductive construction of theory and deductive application of theory are needed.

While the use of theory does not necessarily yield more effective implementation than using common sense, there are certain advantages to applying formal theory over common sense (i.e. informal theory). Theories are explicit and open to question and examination; common sense usually consists of implicit assumptions, beliefs and ways of thinking and is therefore more difficult to challenge. If deductions from a theory are incorrect, the theory can be adapted, extended or abandoned. Theories are more consistent with existing facts than common sense, which typically means that a hypothesis based on an established theory is a more educated guess than one based on common sense. Furthermore, theories give individual facts a meaningful context and contribute towards building an integrated body of knowledge, whereas common sense is more likely to produce isolated facts [145,146]. On the other hand, theory may serve as blinders, as suggested by Kuhn [147] and Greenwald et al. [148], causing us to ignore problems that do not fit into existing theories, models and frameworks or hindering us from seeing known problems in new ways. Theorizing about implementation should therefore not be an abstract academic exercise unconnected with the real world of implementation practice. In the words of Immanuel Kant, “Experience without theory is blind, but theory without experience is mere intellectual play”.

## References

[1] Eccles M, Grimshaw J, Walker A, Johnston M, Pitts N (2005). Changing the behavior of healthcare professionals: the use of theory in promoting the uptake of research findings. J Clin Epidemiol.

[2] Davies P, Walker A, Grimshaw J (2003). Theories of behavior change in studies of guideline implementation. Proc Br Psychol Soc.

[3] Michie S, Johnston M, Abraham C, Lawton R, Parker D, Walker A (2005). Making psychological theory useful for implementing evidence based practice: a consensus approach. Qual Saf Health Care.

[4] Sales A, Smith J, Curran G, Kochevar L (2006). Models, strategies, and tools: theory in implementing evidence-based findings into health care practice. J Gen Intern Med.

[5] Kitson AL, Harvey G, McCormack B (1998). Enabling the implementation of evidence-based practice: a conceptual framework. Qual Health Care.

[6] ICEBeRG (2006). Designing theoretically-informed implementation interventions. Implement Sci.

[7] Godin G, Bélanger-Gravel A, Eccles M, Grimshaw J (2008). Healthcare professionals’ intentions and behaviours: a systematic review of studies based on social cognitive theories. Implement Sci.

[8] Mitchell SA, Fisher CA, Hastings CE, Silverman LB, Wallen GR (2010). A thematic analysis of theoretical models for translating science in nursing: mapping the field. Nurs Outlook.

[9] Rycroft-Malone J, Bucknall T, Rycroft-Malone J, Bucknall T (2010). Theory, Frameworks, and Models: Laying Down the Groundwork. Models and Frameworks for Implementing Evidence-Based Practice: Linking Evidence to Action.

[10] Cane J, O’Connor D, Michie S (2012). Validation of the theoretical domains framework for use in behaviour change and implementation research. Implement Sci.

[11] Martinez RG, Lewis CC, Weiner BJ (2014). Instrumentation issues in implementation science. Implement Sci.

[12] Eccles MP, Mittman BS (2006). Welcome to implementation science. Implement Sci.

[13] Graham ID, Logan J, Harrison MB, Straus SE, Tetroe J, Caswell W (2006). Lost in knowledge translation: time for a map?. J Contin Educ Health Prof.

[14] Estabrooks CA, Thompson DS, Lovely JE, Hofmeyer A (2006). A guide to knowledge translation theory. J Contin Educ Health Prof.

[15] Wilson KM, Brady TJ, Lesesne C, on behalf of the NCCDPHP Work Group on Translation (2011). An organizing framework for translation in public health: the knowledge to action framework. Prev Chronic Dis.

[16] Rabin BA, Brownson RC, Brownson RC, Colditz GA, Proctor EK (2012). Developing the Terminology for Dissemination and Implementation Research. Dissemination and Implementation Research in Health.

[17] Greenhalgh T, Robert G, Bate P, Macfarlane F, Kyriakidou O (2005). Diffusion of Innovations in Service Organisations: A Systematic Literature Review.

[18] Jones K (2004). Mission drift in qualitative research, or moving toward a systematic review of qualitative studies, moving back to a more systematic narrative review. Qual Rep.

[19] Cronin P, Ryan F, Coughlan M (2008). Undertaking a literature review: a step-by-step approach. Br J Nurs.

[20] Rycroft-Malone J, Bucknall T (2010). Models and Frameworks for Implementing Evidence-Based Practice: Linking Evidence to Action.

[21] Nutley SM, Walter I, Davies HTO (2007). Using evidence: how research can inform public services.

[22] Grol R, Wensing M, Eccles M (2005). Improving Patient Care: The Implementation of Change in Clinical Practice.

[23] Straus S, Tetroe J, Graham ID (2009). Knowledge Translation in Health Care.

[24] Brownson RC, Colditz GA, Proctor EK (2012). Dissemination and Implementation Research in Health.

[25] Graham ID, Tetroe J (2007). Some theoretical underpinnings of knowledge translation. Acad Emerg Med.

[26] Flottorp SA, Oxman AD, Krause J, Musila NR, Wensing M, Godycki-Cwirko M (2013). A checklist for identifying determinants of practice: a systematic review and synthesis of frameworks and taxonomies of factors that prevent or enable improvements in healthcare professional practice. Implement Sci.

[27] Meyers DC, Durlak JA, Wandersman A (2012). The Quality Implementation Framework: a synthesis of critical steps in the implementation process. Am J Community Psychol.

[28] Tabak RG, Khoong EC, Chambers DA, Brownson RC (2012). Bridging research and practice: models for dissemination and implementation research. Am J Prev Med.

[29] Frankfort-Nachmias C, Nachmias D (1996). Research Methods in the Social Sciences.

[30] Wacker JG (1998). A definition of theory: research guidelines for different theory-building research methods in operations management. J Oper Manag.

[31] Carpiano RM, Daley DM (2006). A guide and glossary on postpositivist theory building for population health. J Epidemiol Community Health.

[32] Bunge M (1967). Scientific Research 1: The Search for System.

[33] Reynolds PD (1971). A Primer in Theory Construction.

[34] Dubin R (1978). Theory Building.

[35] Hunt SD (1991). Modern Marketing Theory: Critical Issues in the Philosophy of Marketing Science.

[36] Bluedorn AC, Evered RD, Pinder CC, Moore LF (1980). Middle Range Theory and the Strategies of Theory Construction. Middle Range Theory and The Study of Organizations.

[37] Cairney P (2012). Understanding Public Policy—Theories and Issues.

[38] Sabatier PA (2007). Theories of the Policy Process.

[39] Kitson AL, Rycroft-Malone J, Harvey G, McCormack B, Seers K, Titchen A (2008). Evaluating the successful implementation of evidence into practice using the PARiHS framework: theoretical and practical challenges. Implement Sci.

[40] Huberman M (1994). Research utilization: the state of the art. Knowl Policy.

[41] Landry R, Amara N, Lamari M (2001). Climbing the ladder of research utilization: evidence from social science. Sci Commun.

[42] Canadian Institutes of Health Research (CIHR). About knowledge translation. [http://www.cihr-irsc.gc.ca/e/29418.html]. Retrieved 18 December 2014.

[43] Davis SM, Peterson JC, Helfrich CD, Cunningham-Sabo L (2007). Introduction and conceptual model for utilization of prevention research. Am J Prev Med.

[44] Majdzadeh R, Sadighi J, Nejat S, Mahani AS, Ghdlami J (2008). Knowledge translation for research utilization: design of a knowledge translation model at Teheran University of Medical Science. J Contin Educ Health Prof.

[45] Graham ID, Tetroe J, Straus S, Tetroe J, Graham ID, KT Theories Group (2009). Planned Action Theories. Knowledge Translation in Health Care.

[46] Rycroft-Malone J, Bucknall T, Rycroft-Malone J, Bucknall T (2010). Analysis and Synthesis of Models and Frameworks. Models and Frameworks for Implementing Evidence-Based Practice: Linking Evidence to Action.

[47] Stetler CB, Rycroft-Malone J, Bucknall T (2010). Stetler model. Models and Frameworks for Implementing Evidence-Based Practice: Linking Evidence to Action.

[48] Stevens KR (2013). The impact of evidence-based practice in nursing and the next big ideas. Online J Issues Nurs.

[49] Titler MG, Kleiber C, Steelman V, Goode C, Rakel B, Barry-Walker J (1995). Infusing research into practice to promote quality care. Nurs Res.

[50] Titler MG, Kleiber C, Steelman VJ, Rakel BA, Budreau G, Everett LQ (2001). The Iowa Model of evidence-based practice to promote quality care. Crit Care Nurs Clin North Am.

[51] Logan J, Graham I (1998). Toward a comprehensive interdisciplinary model of health care research use. Sci Commun.

[52] Logan J, Graham I, Rycroft-Malone J, Bucknall T (2010). The Ottawa Model of Research Use. Models and Frameworks for Implementing Evidence-Based Practice: Linking Evidence to Action.

[53] Grol R, Wensing M (2004). What drives change? Barriers to and incentives for achieving evidence-based practice. Med J Aust.

[54] Pronovost PJ, Berenholtz SM, Needham DM (2008). Translating evidence into practice: a model for large scale knowledge translation. BMJ.

[55] Field B, Booth A, Ilott I, Gerrish K (2014). Using the Knowledge to Action Framework in practice: a citation analysis and systematic review. Implement Sci.

[56] Stetler C (1994). Refinement of the Stetler/Marram Model for application of research findings to practice. Nurs Outlook.

[57] Durlak JA, DuPre EP (2008). Implementation matters: a review of research on the influence of implementation on program outcomes and the factors affecting implementation. Am J Community Psychol.

[58] Gurses AP, Marsteller JA, Ozok AA, Xiao Y, Owens S, Pronovost PJ (2010). Using an interdisciplinary approach to identify factors that affect clinicians’ compliance with evidence-based guidelines. Crit Care Med.

[59] Cochrane LJ, Olson CA, Murray S, Dupuis M, Tooman T, Hayes S (2007). Gaps between knowing and doing: understanding and assessing the barriers to optimal health care. J Contin Educ Health Prof.

[60] Damschroder LJ, Aron DC, Keith RE, Kirsh SR, Alexander JA, Lowery JC (2009). Fostering implementation of health services research findings into practice: a consolidated framework for advancing implementation science. Implement Sci.

[61] Ferlie E, Shortell SM (2001). Improving the quality of health care in the United Kingdom and the United States: a framework for change. Milbank Q.

[62] Jacobson N, Butterill D, Goering P (2003). Development of a framework for knowledge translation: understanding user context. J Health Serv Res Policy.

[63] Blase KA, Van Dyke M, Fixsen DL, Bailey FW, Kelly B, Perkins DF (2012). Implementation Science: Key Concepts, Themes and Evidence for Practitioners in Educational Psychology. Handbook of Implementation Science for Psychology in Education.

[64] Rycroft-Malone J, Rycroft-Malone J, Bucknall T (2010). Promoting Action on Research Implementation in Health Services (PARIHS). Models and Frameworks for Implementing Evidence-Based Practice: Linking Evidence to Action.

[65] Helfrich CD, Damschroder LJ, Hagedorn HJ, Daggett GS, Sahay A, Ritchie M (2010). A critical synthesis of literature on the Promoting Action on Research Implementation in Health Services (PARIHS) framework. Implement Sci.

[66] Michie S, Atkins L, West R (2014). A guide to using the Behaviour Change Wheel.

[67] Fixsen DL, Naoom SF, Blase KA, Friedman RM, Wallace F (2005). Implementation Research: A Synthesis of the Literature.

[68] Holmes BJ, Finegood DT, Riley BL, Best A, Brownson RC, Colditz GA, Proctor EK (2012). Systems Thinking in Dissemination and Implementation Research. Dissemination and Implementation Research in Health.

[69] Légaré F, Ratté S, Gravel K, Graham ID (2008). Barriers and facilitators to implementing shared decision-making in clinical practice. Update of a systematic review of health professionals’ perceptions. Patient Educ Couns.

[70] Johnson M, Jackson R, Guillaume L, Meier P, Goyder E (2011). Barriers and facilitators to implementing screening and brief intervention for alcohol misuse: a systematic review of qualitative evidence. J Public Health.

[71] Verweij LM, Proper KI, Leffelaar ER, Weel ANH, Nauta AP, Hulshof CTJ (2012). Barriers and facilitators to implementation of an occupational health guideline aimed at preventing weight gain among employees in the Netherlands. J Occup Environ Med.

[72] Broyles LM, Rodriguez KL, Kraemer KL, Sevick MA, Price PA, Gordon AJ (2012). A qualitative study of anticipated barriers and facilitators to the implementation of nurse-delivered alcohol screening, brief intervention, and referral to treatment for hospitalized patients in a Veterans Affairs medical centre. Addiction Sci Clin Pract.

[73] Dopson S, Fitzgerald L, Dopson S, Fitzgerald L (2005). The Active Role of Context. Knowledge to Action? Evidence-Based Health Care in Context.

[74] Ashton CM, Khan MM, Johnson ML, Walder A, Stanberry E, Beyth RJ (2007). A quasi-experimental test of an intervention to increase the use of thiazide-based treatment regimens for people with hypertension. Implement Sci.

[75] Mohr DC, VanDeusen LC, Meterko M (2008). Predicting healthcare employees’ participation in an office redesign program: attitudes, norms and behavioral control. Implement Sci.

[76] Scott SD, Plotnikoff RC, Karunamuni N, Bize R, Rodgers W (2008). Factors influencing the adoption of an innovation: an examination of the uptake of the Canadian Heart Health Kit (HHK). Implement Sci.

[77] Zardo P, Collie A (2014). Predicting research use in a public health policy environment: results of a logistic regression analysis. Implement Sci.

[78] Gabbay J, Le May A (2011). Evidence based guidelines or collectively constructed “mindlines”? Ethnographic study of knowledge management in primary care. Br Med J.

[79] Fishbein M, Ajzen I (1975). Belief, Attitude, Intention, and Behaviour.

[80] Bandura A (1977). Self-efficacy: towards a unifying theory of behavioural change. Psychol Rev.

[81] Bandura A (1986). Social Foundations of Thought and Action: A Cognitive Social Theory.

[82] Triandis HC (1979). Values, Attitudes, and Interpersonal Behaviour. Nebraska Symposium on Motivation; Beliefs, Attitude, and values: 1979.

[83] Ajzen I (1988). Attitudes, Personality and Behavior.

[84] Nilsen P, Roback K, Broström A, Ellström PE (2012). Creatures of habit: accounting for the role of habit in implementation research on clinical behaviour change. Implement Sci.

[85] Hammond KR (1981). Principles of Organization in Intuitive and Analytical Cognition.

[86] Benner P (1984). From Novice to Expert, Excellence and Power in Clinical Nursing Practice.

[87] Epstein S (1994). Integration of the cognitive and the psychodynamic unconscious. Am Psychol.

[88] Ouelette JA, Wood W (1998). Habit and intention in everyday life: the multiple processes by which past behaviour predicts future behaviour. Psychol Bull.

[89] Verplanken B, Aarts H (1999). Habit, attitude, and planned behaviour: is habit an empty construct or an interesting case of goal-directed automaticity?. Eur Rev Soc Psychol.

[90] Eccles MP, Hrisos S, Francis JJ, Steen N, Bosch M, Johnston M (2009). Can the collective intentions of individual professionals within healthcare teams predict the team’s performance: developing methods and theory. Implement Sci.

[91] Parchman ML, Scoglio CM, Schumm P (2011). Understanding the implementation of evidence-based care: a structural network approach. Implement Sci.

[92] Cunningham FC, Ranmuthugala G, Plumb J, Georgiou A, Westbrook JI, Braithwaite J. Health professional networks as a vector for improving healthcare quality and safety: a systematic review. BMJ Qual Saf. 2011. doi:10.1136/bmjqs-2011-000187. 10.1136/bmjqs-2011-000187 PMC3285140 22129933

[93] Mascia D, Cicchetti A (2011). Physician social capital and the reported adoption of evidence-based medicine: exploring the role of structural holes. Soc Sci Med.

[94] Wallin L, Ewald U, Wikblad K, Scott-Findlay S, Arnetz BB (2006). Understanding work contextual factors: a short-cut to evidence-based practice?. Worldviews Evid Based Nurs.

[95] Meijers JMM, Janssen MAP, Cummings GG, Wallin L, Estabrooks CA, Halfens RYG (2006). Assessing the relationship between contextual factors and research utilization in nursing: systematic literature review. J Adv Nurs.

[96] Wensing M, Wollersheim H, Grol R (2006). Organizational interventions to implement improvements in patient care: a structured review of reviews. Implement Sci.

[97] Gifford W, Davies B, Edwards N, Griffin P, Lybanon V (2007). Managerial leadership for nurses’ use of research evidence: an integrative review of the literature. Worldviews Evid Based Nurs.

[98] Yano EM (2008). The role of organizational research in implementing evidence-based practice: QUERI series. Implement Sci.

[99] French B, Thomas LH, Baker P, Burton CR, Pennington L, Roddam H (2009). What can management theories offer evidence-based practice? A comparative analysis of measurement tools for organizational context. Implement Sci.

[100] Parmelli E, Flodgren G, Beyer F, Baillie N, Schaafsma ME, Eccles MP (2011). The effectiveness of strategies to change organisational culture to improve healthcare performance: a systematic review. Implement Sci.

[101] Chaudoir SR, Dugan AG, Barr CHI (2013). Measuring factors affecting implementation of health innovations: a systematic review of structural, organizational, provider, patient, and innovation level measures. Implement Sci.

[102] Orlikowski W (1994). Improvising organizational transformation over time: a situated change perspective. Inform Syst Res.

[103] DiMaggio PJ, Powell WW (1991). The New Institutionalism and Organizational Analysis.

[104] Scott WR (1995). Institutions and Organizations.

[105] Plsek PE, Greenhalgh T (2001). The challenge of complexity in health care. BMJ.

[106] Waldrop MM (1992). Complexity: The Emerging Science at The Edge of Order and Chaos.

[107] Rogers EM (2003). Diffusion of Innovations.

[108] Aubert BA, Hamel G (2001). Adoption of smart cards in the medical sector: the Canadian experience. Soc Sci Med.

[109] Vollink T, Meertens R, Midden CJH (2002). Innovating ‘diffusion of innovation’ theory: innovation characteristics and the intention of utility companies to adopt energy conservation interventions. J Environ Psychol.

[110] Foy R, MacLennan G, Grimshaw J, Penney G, Campbell M, Grol R (2002). Attributes of clinical recommendations that influence change in practice following audit and feedback. J Clin Epidemiol.

[111] Oxman AD, Thomson MA, Davis DA, Haynes RB (1995). No magic bullets: a systematic review of 102 trials of interventions to improve professional practice. CMAJ.

[112] Grimshaw J, McAuley LM, Bero LA, Grilli R, Oxman AD, Ramsay C (2003). Systematic reviews of effectiveness of quality improvement strategies and programmes. Qual Saf Health Care.

[113] Walter I, Nutley SM, Davies HTO. Developing a taxonomy of interventions used to increase the impact of research. St. Andrews: University of St Andrews; 2003. Discussion Paper 3, Research Unit for Research Utilisation, University of St. Andrews.

[114] Leeman J, Baernholdt M, Sandelowski M (2007). Developing a theory-based taxonomy of methods for implementing change in practice. J Adv Nurs.

[115] Estabrooks CA, Derksen L, Winther C, Lavis JN, Scott SD, Wallin L (2008). The intellectual structure and substance of the knowledge utilization field: a longitudinal author co-citation analysis, 1945 to 2004. Implement Sci.

[116] Klein KJ, Sorra JS (1996). The challenge of innovation implementation. Acad Manage Rev.

[117] Zahra AS, George G (2002). Absorptive capacity: a review, reconceptualization and extension. Acad Manage Rev.

[118] Weiner BJ (2009). A theory of organizational readiness for change. Implement Sci.

[119] Michie S, van Stralen MM, West R (2011). The behaviour change wheel: a new method for characterising and designing behaviour change interventions. Implement Sci.

[120] May C, Finch T (2009). Implementing, embedding and integrating practices: an outline of Normalization Process Theory. Sociology.

[121] May C, Finch T, Mair F, Ballini L, Dowrick C, Eccles M (2007). Understanding the implementation of complex interventions in health care: the normalization process model. Implement Sci.

[122] Finch TL, Rapley T, Girling M, Mair FS, Murray E, Treweek S (2013). Improving the normalization of complex interventions: measure development based on normalization process theory (NoMAD): study protocol. Implement Sci.

[123] Murray E, Treweek S, Pope C, MacFarlane A, Ballini L, Dowrick C (2010). Normalisation process theory: a framework for developing, evaluating and implementing complex interventions. BMC Med.

[124] Glasgow RE, Vogt TM, Boles SM (1999). Evaluating the public health impact of health promotion interventions: the RE-AIM framework. Am J Public Health.

[125] Green LW, Kreuter MW (2005). Health Program Planning: An Educational and Ecological Approach.

[126] Proctor E, Silmere H, RaghaVan R, Hovmand P, Aarons G, Bunger A (2011). Outcomes for implementation research: conceptual distinctions, measurement challenges, and research agenda. Admin Policy Mental Health.

[127] Phillips CJ, Marshall AP, Chaves NJ, Lin IB, Loy CT, Rees G (2015). Experiences of using Theoretical Domains Framework across diverse clinical environments: a qualitative study. J Multidiscip Healthc.

[128] Fleming A, Bradley C, Cullinan S, Byrne S (2014). Antibiotic prescribing in long-term care facilities: a qualitative, multidisciplinary investigation. BMJ Open.

[129] McEvoy R, Ballini L, Maltoni S, O’Donnell CA, Mair FS, MacFarlane A (2014). A qualitative systematic review of studies using the normalization process theory to research implementation processes. Implement Sci.

[130] Connell LA, McMahon NE, Redfern J, Watkins CL, Eng JJ (2015). Development of a behaviour change intervention to increase upper limb exercise in stroke rehabilitation. Implement Sci.

[131] Praveen D, Patel A, Raghu A, Clifford GD, Maulik PK, Abdul AM (2014). Development and field evaluation of a mobile clinical decision support system for cardiovascular diseases in rural India. JMIR mHealth uHealth.

[132] Estabrooks CA, Squires JE, Cummings GG, Birdell JM, Norton PG (2009). Development and assessment of the Alberta Context Tool. BMC Health Serv Res.

[133] McCormack B, McCarthy G, Wright J, Slater P, Coffey A (2009). Development and testing of the Context Assessment Index (CAI). Worldviews Evid Based Nurs.

[134] Damschroder LJ, Lowery JC (2013). Evaluation of a large-scale weight management program using the consolidated framework for implementation research (CFIR). Implement Sci.

[135] Dyson J, Lawton R, Jackson C, Cheater F (2013). Development of a theory-based instrument to identify barriers and levers to best hand hygiene practice among healthcare practitioners. Implement Sci.

[136] Melnyk BM, Fineout-Overholt E, Mays MZ (2008). The Evidence-Based Practice Beliefs and Implementation Scales: psychometric properties of two new instruments. Worldviews Evid Based Nurs.

[137] Nilsson Kajermo K, Boström A-M, Thompson DS, Hutchinson AM, Estabrooks CA, Wallin L (2010). The BARRIERS scale—the barriers to research utilization scale: a systematic review. Implement Sci.

[138] Jacobs SR, Weiner BJ, Bunger AC (2014). Context matters: measuring implementation climate among individuals and groups. Implement Sci.

[139] Gagnon M-P, Labarthe J, Légaré F, Ouimet M, Estabrooks CA, Roch G (2011). Measuring organizational readiness for knowledge translation in chronic care. Implement Sci.

[140] Osigweh CAB (1989). Concept fallibility in organizational science. Acad Manage Rev.

[141] May C (2013). Towards a general theory of implementation. Implement Sci.

[142] Michie S, Abraham C, Eccles MP, Francis JJ, Hardeman W, Jonston M (2011). Strengthening evaluation and implementation by specifying components of behaviour change interventions: a study protocol. Implement Sci.

[143] Oxman AD, Fretheim A, Flottorp S (2005). The OFF theory of research utilization. J Clin Epidemiol.

[144] Bhattacharyya O, Reeves S, Garfinkel S, Zwarenstein M (2006). Designing theoretically-informed implementation interventions: fine in theory, but evidence of effectiveness in practice is needed. Implement Sci.

[145] Fletcher GJO (1984). Psychology and common sense. Am Psychol.

[146] Cacioppo JT (2004). Common sense, intuition and theory in personality and social psychology. Pers Soc Psychol Rev.

[147] Kuhn TS (1970). The Structure of Scientific Revolutions.

[148] Greenwald AG, Pratkanis AR, Leippe MR, Baumgardner MH (1986). Under what conditions does theory obstruct research progress?. Psychol Rev.

